# Oral delivery of *Lactococcus lactis* that secretes bioactive heme oxygenase-1 alleviates development of acute colitis in mice

**DOI:** 10.1186/s12934-015-0378-2

**Published:** 2015-11-25

**Authors:** Suguru Shigemori, Takafumi Watanabe, Kai Kudoh, Masaki Ihara, Shireen Nigar, Yoshinari Yamamoto, Yoshihito Suda, Takashi Sato, Haruki Kitazawa, Takeshi Shimosato

**Affiliations:** Department of Bioscience and Food Production Science, Interdisciplinary Graduate School of Science and Technology, Shinshu University, 8304 Minamiminowa, Kamiina, Nagano, 399-4598 Japan; Research Fellow of the Japan Society for the Promotion of Science, Japan Society for the Promotion of Science (JSPS), 5-3-1, Kojimachi, Chiyoda-ku, Tokyo, 102-0083 Japan; Department of Food Production Science, Graduate School of Agriculture, Shinshu University, 8304 Minamiminowa, Kamiina, Nagano, 399-4598 Japan; Department of Bioscience and Biotechnology, Graduate School of Agriculture, Shinshu University, 8304 Minamiminowa, Kamiina, Nagano, 399-4598 Japan; Department of Interdisciplinary Genome Sciences and Cell Metabolism, Institute for Biomedical Sciences (IBS), Interdisciplinary Cluster for Cutting Edge Research (ICCER), Shinshu University, 8304 Minamiminowa, Kamiina, Nagano, 399-4598 Japan; Department of Food, Agriculture and Environment, Miyagi University, 2-2-1 Hatadate, Taihaku-ku, Sendai, Miyagi 982-0215 Japan; Department of Internal Medicine and Clinical Immunology, Graduate School of Medicine, Yokohama City University, 3-9 Fukuura, Kanazawa-ku, Yokohama, Kanagawa 236-0004 Japan; Food and Feed Immunology Group, Graduate School of Agricultural Science, Tohoku University, 1-1 Tsutsumidori-Amamiyamachi, Aoba-ku, Sendai, Miyagi 981-8555 Japan; Livestock Immunology Unit, International Education and Research Center for Food and Agricultural Immunology (CFAI), Graduate School of Agricultural Science, Tohoku University, 1-1 Tsutsumidori-Amamiyamachi, Aoba-ku, Sendai, Miyagi 981-8555 Japan; Department of Sciences of Functional Foods, Graduate School of Agriculture, Shinshu University, 8304 Minamiminowa, Kamiina, Nagano, 399-4598 Japan

**Keywords:** *Lactococcus lactis*, Heme oxygenase-1, Acute colitis, Interleukin-10, Mice, Inflammatory bowel disease

## Abstract

**Background:**

Mucosal delivery of therapeutic proteins using genetically modified strains of lactic acid bacteria (gmLAB) is being investigated as a new therapeutic strategy.

**Methods:**

We developed a strain of gmLAB, *Lactococcus lactis* NZ9000 (NZ-HO), which secretes the anti-inflammatory molecule recombinant mouse heme oxygenase-1 (rmHO-1). The effects of short-term continuous oral dosing with NZ-HO were evaluated in mice with dextran sulfate sodium (DSS)-induced acute colitis as a model of inflammatory bowel diseases (IBD).

**Results:**

We identified the secretion of rmHO-1 by NZ-HO. rmHO-1 was biologically active as determined with spectroscopy. Viable NZ-HO was directly delivered to the colon via oral administration, and rmHO-1 was secreted onto the colonic mucosa in mice. Acute colitis in mice was induced by free drinking of 3 % DSS in water and was accompanied by an increase in the disease activity index score and histopathological changes. Daily oral administration of NZ-HO significantly improved these colitis-associated symptoms. In addition, NZ-HO significantly increased production of the anti-inflammatory cytokine interleukin (IL)-10 and decreased the expression of pro-inflammatory cytokines such as IL-1α and IL-6 in the colon compared to a vector control strain.

**Conclusions:**

Oral administration of NZ-HO alleviates DSS-induced acute colitis in mice. Our results suggest that NZ-HO may be a useful mucosal therapeutic agent for treating IBD.

## Background

Inflammatory bowel diseases (IBD) including ulcerative colitis and Crohn’s disease are chronic inflammatory disorders of the gastrointestinal tract in humans, and they constitute an important global public health problem with increasing incidence [1]. However, the exact etiology of these disorders remains unclear [2], and current IBD therapies with corticosteroids and monoclonal antibodies are not effective because many patients do not respond to these treatments and experience severe side effects [3, 4]. Therefore, novel therapeutic strategies are required to treat and prevent IBD. Recently, many studies have suggested that oral delivery of anti-inflammatory molecules to the intestinal mucosa using genetically modified strains of lactic acid bacteria (gmLAB) may be an alternative strategy for prophylaxis and treatment of inflammatory disorders in the gastrointestinal tract [5–11]. A gmLAB strain expressing a heterologous protein has recently emerged as a safe, effective, and low-cost vehicle for the delivery of bioactive proteins to the intestinal mucosa [12].

The anti-inflammatory and cell protective activities of heme oxygenase-1 (HO-1) have been established in several models [13]. HO-1 is a rate-limiting enzyme in heme catabolism, which leads to the generation of biliverdin, free iron, and carbon monoxide (CO) [14]. Physiologically, HO-1 is an enzyme which can be induced by a number of stimuli, such as inflammatory stimuli and oxidative stress [15–17], and the catabolites produced by the HO-1 reaction exert anti-inflammatory and cytoprotective functions [13]. Previous studies suggested that HO-1 exerts a protective function against intestinal inflammation [18, 19]. Furthermore, in chemically-induced and genetic IBD rodent models, administration of HO-1 inducers alleviates colitis through various mechanisms including suppression of oxidative, pro-apoptotic, and pro-inflammatory responses, as well as facilitation of anti-inflammatory responses and bacterial clearance [18–24].

In this study, we hypothesized that the direct delivery of HO-1 using gmLAB may be a valuable strategy for the treatment of IBD. We explored whether HO-1-secreting *L. lactis* plays a role in attenuating dextran sulfate sodium (DSS)-induced acute colitis. We first engineered a strain of *L. lactis* that secretes recombinant mouse HO-1 (rmHO-1) and evaluated whether rmHO-1 is a bioactive protein. To clarify the role of rmHO-1-secreting *L. lactis* (NZ-HO) through oral administration, we assessed the expression of rmHO-1 in colonic mucosa and the effects in an acute colitis model (IBD model). The results of our study suggest that NZ-HO is a potent anti-inflammatory modulator and therefore may be effective as a mucosal therapeutic agent.

## Results

### Production and secretion of rmHO-1 by NZ-HO

**Fig. 1:**
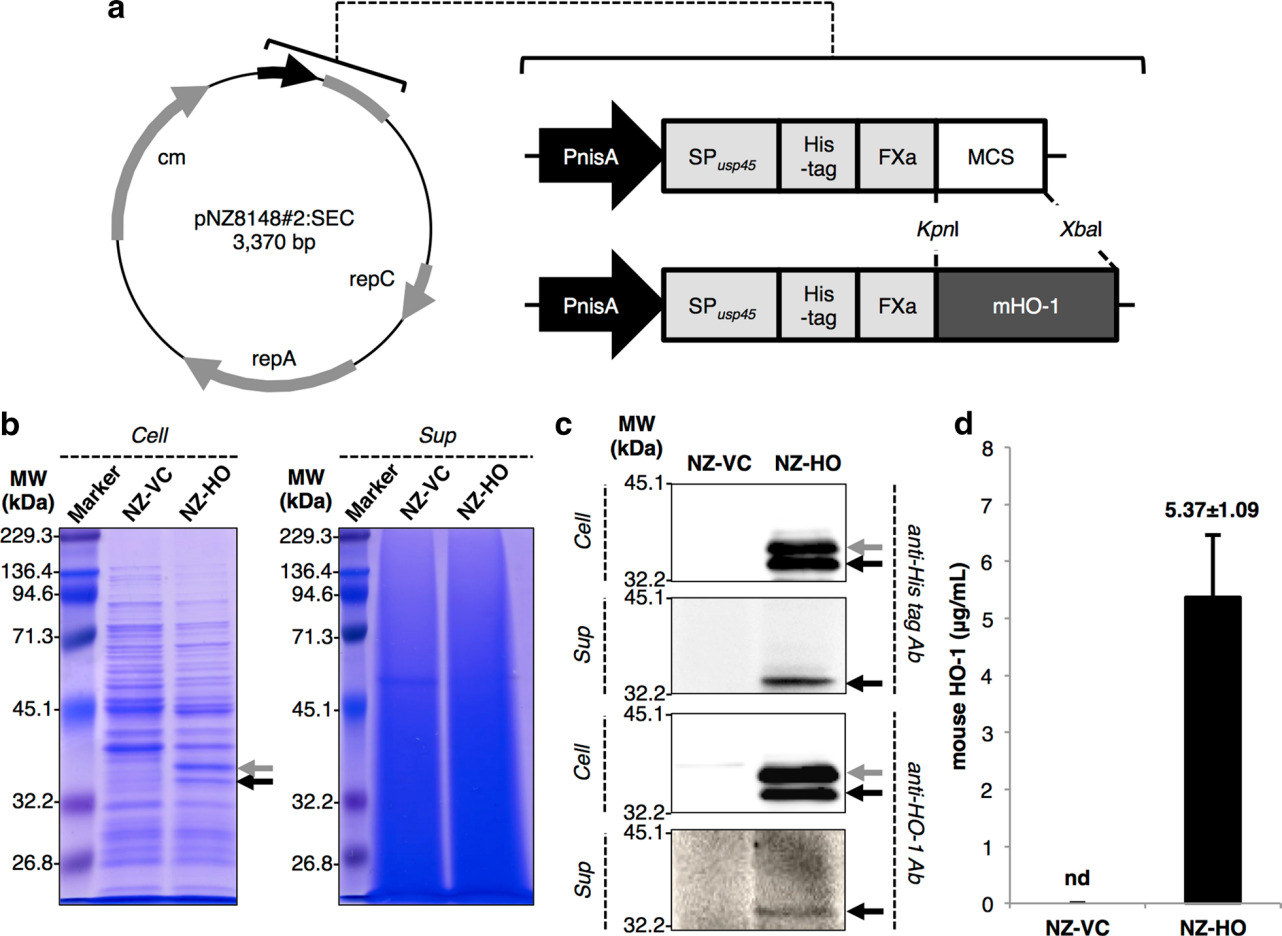
Detection and quantification of rmHO-1 in NZ-HO. **a** A vector map of the lactococcal secretion vector, pNZ8148#2:SEC (*left*), and schematic representations of gene maps from P_*nis*_ to the MCS of pNZ8148#2:SEC (*right*). *P*_*nis*_ nisin-inducible promoter, *SP*_*usp45*_ sequence of the signal peptide from the USP45 protein, *His-tag* 6 × histidine-tag, *FXa* factor Xa recognition site, *MCS* multiple cloning site, *rep* replication gene, *cm* chloramphenicol acetyltransferase gene, *mHO-1* mouse heme oxygenase-1. **b**, **c** Gene expression was induced in gmNZ9000 with nisin. Cellular extracts (*Cell*) and culture supernatants (*Sup*) were analyzed with SDS-PAGE (**b**) or western blotting using an anti-His-tag Ab or anti-HO-1 Ab. *MW* indicates the molecular mass markers (kDa). *Gray* and *black arrows* indicate the precursor of secreted rmHO-1 (pre-rmHO-1, 40.5 kDa) and the secreted form of rmHO-1 (rmHO-1, 37.7 kDa), respectively. Representative images from three independent experiments are shown. **d** mHO-1 in cellular extracts from nisin-induced gmNZ9000 was quantified by ELISA. Data are the mean ± SD (*n* = 6). *nd* not detected

We constructed a mHO-1 secretion vector. Gene expression of rmHO-1, which was conjugated to a lactococcal signal peptide and a 6 × histidine (His) tag, was controlled by a nisin-inducible promoter (P_*nis*_) (Fig. 1a). The resulting plasmid was introduced into *L. lactis* NZ9000. SDS-PAGE (Fig. 1b) and western blotting (Fig. 1c) using anti-His tag antibody (Ab) and anti-HO-1 Ab showed bands corresponding to the secreted precursor of rmHO-1 (pre-rmHO-1, 40.5 kDa) and the secreted form of rmHO-1 (rmHO-1, 37.7 kDa) in cellular extracts of nisin-induced NZ-HO. Western blotting showed that only one band corresponding to rmHO-1 was observed in the culture supernatant from NZ-HO (Fig. 1c). These results demonstrated that NZ-HO intracellularly expresses pre-rmHO-1 upon nisin stimulation, followed by extracellular secretion by the cell’s secretory machinery. An enzyme-linked immunosorbent assay (ELISA) showed that cellular extracts from nisin-induced NZ-HO contained approximately 5 μg/mL mHO-1 (Fig. 1d). No production or secretion of rmHO-1 was observed in the nisin-induced NZ-vector control (NZ-VC) (Fig. 1b–d).

### Bioactivity assay for rmHO-1

**Fig. 2:**
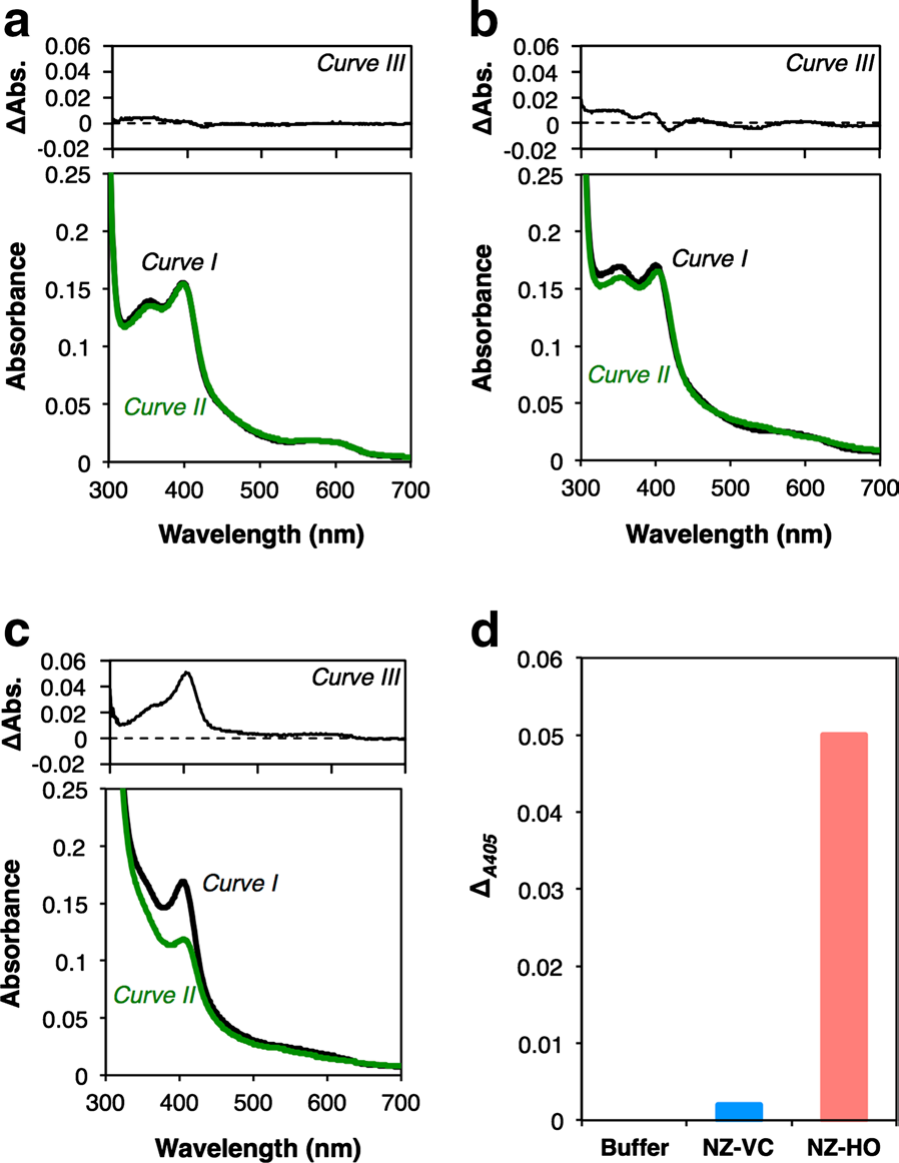
Bioactivity assay of rmHO-1. The absorption spectra of the reaction mixtures containing Kpi buffer (**a**) and cellular extracts from NZ-VC (**b**) or NZ-HO (**c**) were measured at the starting point of the assay (A_*0min*_, *Curve I*) and after 30 min of incubation at 23 °C (A_*30min*_, *Curve II*). The differential spectra (A_*0min*_ − A_*30min*_, *Curve III*) are presented in the *upper graph* of each panel. **d** The absorbance changes at 405 nm are shown. Similar results were obtained from three independent experiments, and representative data are shown

The bioactivity of rmHO-1 produced by NZ-HO was measured spectrophotometrically in the HO reaction system with ascorbic acid as a reducing agent [25]. At the start of the reaction (i.e., just after the addition of heme), the Soret band of the heme molecules was observed around 400 nm in the reaction mixtures containing potassium phosphate (KPi) buffer (*Curve I* in Fig. 2a) or cellular extract from NZ-VC (*Curve I* in Fig. 2b). An absorption peak of a mixture containing extract from NZ-HO was detected at 405 nm (*Curve I* in Fig. 2c), which corresponds to the peak of the heme-HO complex [26]. After 30 min of incubation, the absorption spectra of each mixture stabilized to those seen in *Curve II* in Fig. 2a–c. In the reaction mixture containing NZ-HO extract, the absorption peak at 405 nm was remarkably decreased (*Curve III* in Fig. 2c, d) and became a smaller peak at 400 nm (*Curve II* in Fig. 2c). No considerable change was detected in mixtures containing buffer (*Curve III* in Fig. 2a, d) or NZ-VC extract (*Curve III* in Fig. 2b, d). These results suggest that rmHO-1 formed an enzyme-substrate complex and degraded the heme molecules in the presence of ascorbic acid [25, 26].

### NZ-HO reaches the colonic mucosa following oral administration

**Fig. 3:**
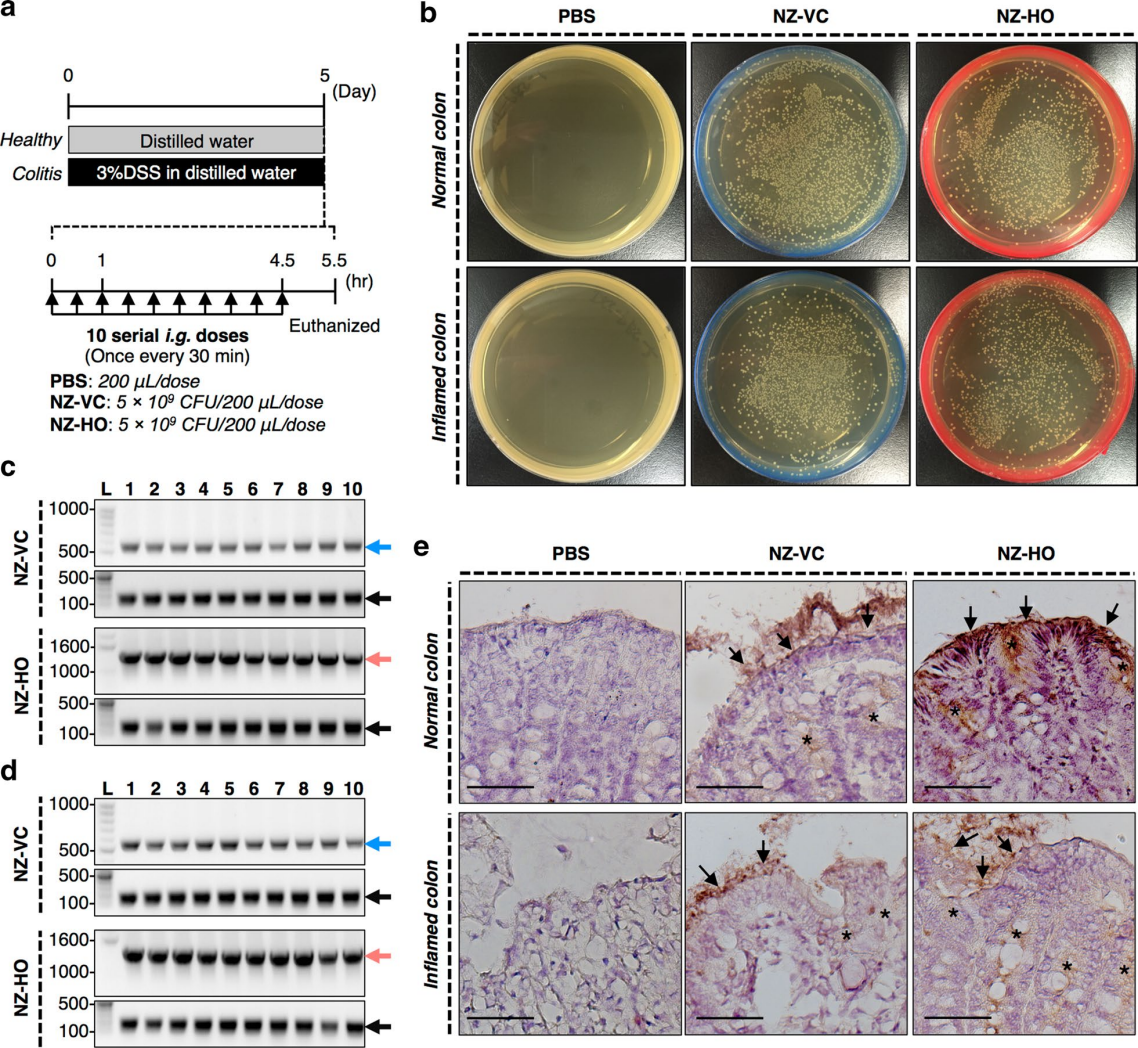
Survival of NZ-HO in the mouse intestine. **a** Experimental schedule. **b** Homogenates of the entire colon (including luminal contents) were plated on GM17 cm agar. **c**, **d** Ten single colonies were randomly chosen for each sample from healthy (**c**) or colitis (**d**) mice and were subjected to colony-direct PCR with pNZ8148-specific (*upper images* of each group) or *L. lactis* subsp. *cremoris*-specific (*lower images* of each group) primer pairs. Bands indicated by *blue* (586 bp), *salmon pink* (1,403 bp), and *black arrows* (163 bp) were further analyzed by DNA sequencing and were consistent with the putative sequences. *L*: DNA ladder (bp). **e** Immunohistochemical staining/detection of His-tagged proteins in colonic tissue. Positive reactions were observed in the mucosal epithelial cells (*arrows*), in the crypt, and the lamina propria (*asterisks*) of the colon from gmNZ9000-treated mice. *Bars* 50 μm. *Normal colon* and *Inflamed colon* mean colons from healthy (mice that drank sterile water) or colitis (mice that drank DSS in the water) mice, respectively. Similar results were seen in two different mice. Representative images are shown

To investigate whether NZ-HO can deliver rmHO-1 to normal or inflamed colonic mucosa, healthy mice or mice treated with DSS to induce colitis were orally administered 10 serial doses of phosphate-buffered saline (PBS), NZ-VC, or NZ-HO (Fig. 3a). Using homogenates of the healthy and inflamed colon from mice given NZ-VC or NZ-HO, growth of colonies of a genetically modified strain of NZ9000 (gmNZ9000) was observed in M17 agar plates supplemented with glucose and chloramphenicol (GM17 cm) (Fig. 3b). In contrast, no colonies were observed when using homogenates from the colon of PBS-treated mice. To characterize the colonies obtained from gmNZ9000-treated healthy (Fig. 3c) and inflamed colons (Fig. 3d), ten randomly selected single colonies from each group were analyzed by the colony-direct polymerase chain reaction (PCR) using two primer pairs: (1) *L. lactis* subsp. *cremoris*-specific primer pair, which hybridizes with the 16S ribosomal DNA on *L. lactis* NZ9000 [27]; and (2) pNZ8148-specific primer pair, which amplifies the region of the nisin-inducible promoter to the tail of the multiple cloning site of pNZ8148#2. Products of 163 bp were amplified in all colonies with the *L. lactis* subsp. *cremoris*-specific primer pair. When the pNZ8148-specific primer pair was used, PCR products representing pNZ8148#2:SEC (586 bp) and pNZ8148#2:SEC-mHO-1 (1403 bp) were obtained from the colonies of NZ-VC and NZ-HO groups, respectively. These products were analyzed by DNA sequencing and were consistent with the putative sequences (data not shown). These results clearly demonstrate that viable NZ-HO reached the colon following oral administration.

We investigated rmHO-1 expression in the colonic mucosa with immunohistochemistry (Fig. 3e). In both healthy mice and mice with an inflamed colon that were treated with gmNZ9000, immunoreactivity of the His-tagged proteins was observed in the mucosal epithelial cells, the crypt and the lamina propria (Fig. 3e). No immunoreactivity was detected in the colonic mucosa of PBS-treated mice.

### Effect of oral administration of NZ-HO to IBD mice

**Fig. 4:**
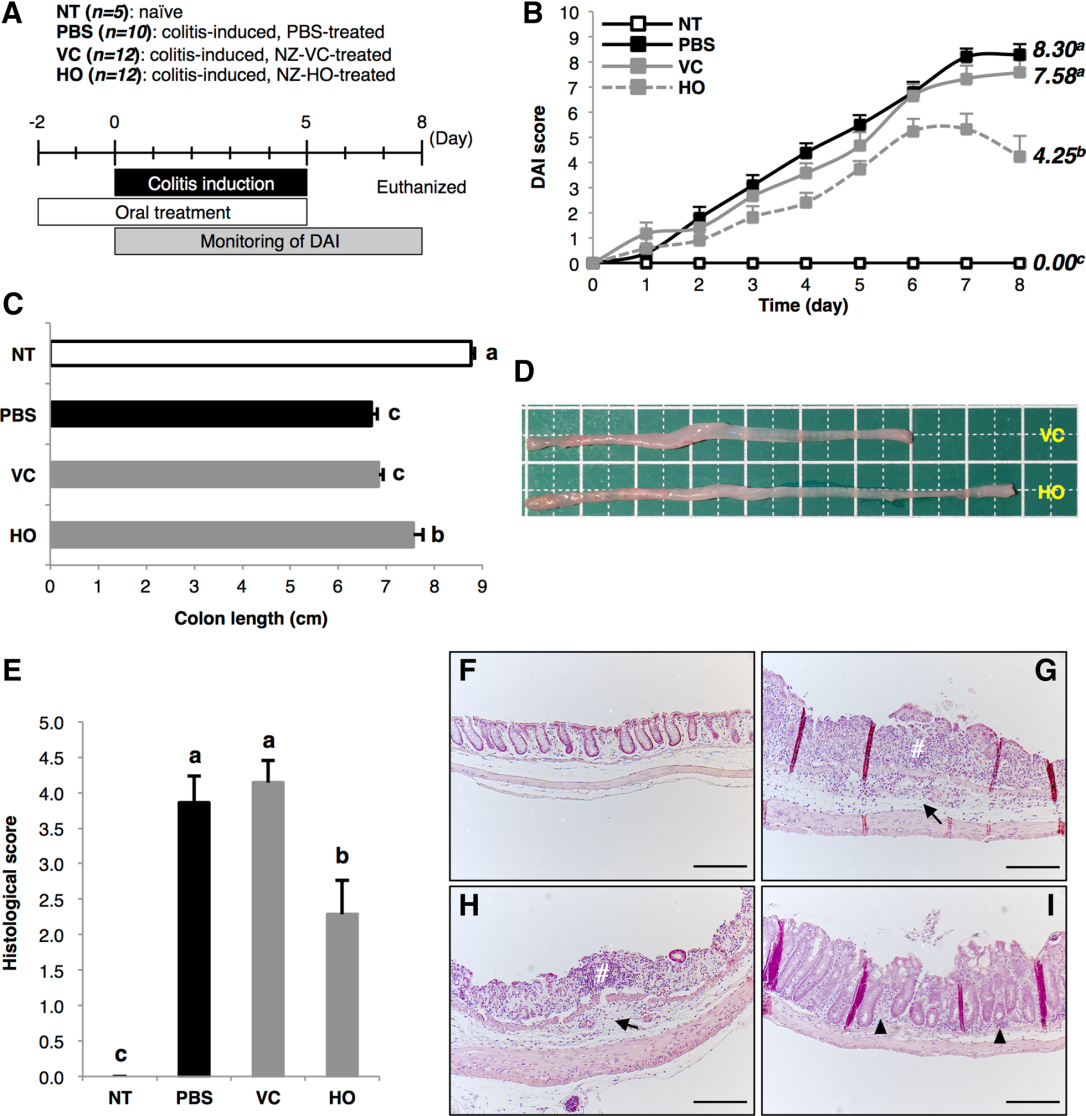
Effects of oral administration of NZ-HO on DSS-induced acute colitis in mice. **A** Experimental schedule for the in vivo experiment. **B** Chronological change in the DAI score (“Methods”) of mice in each group. Data are the mean + SE (*n* = 5–12). **C** On day 8, the colon length (*i.e.*, just under the cecum to the anus) was measured. Data are the mean + SE (*n* = 5–12). **D** Representative images of the colon from the VC and HO groups are shown. *Bars* 1 cm. **E** Histogram of histological scores on day 8. Data are the mean + SE (*n* = 5–7). **F**–**I** Representative images of HE-stained colonic sections indicating the average DAI for each group are shown for the NT (**F**), PBS (**G**), VC (**H**), and HO (**I**) groups. In the PBS and VC groups, inflammatory cell infiltration into the submucosa (*arrows*) and severe tissue damage (i.e., erosion and ulcers, *hash*) were observed. An increase in the number of inflammatory cells in the lamina propria was observed in the HO group (*arrowhead*). *Bars* 100 μm. *Letters* (*i.e.,* a, b, c) represent significant differences (*P* < 0.01)

From the first day of DSS exposure (day 0) to the last day of the experiment (day 8), body weight loss, fecal bleeding, and stool consistency of each mouse were monitored and scored daily as macroscopic markers of symptoms, and the disease activity index (DAI) score was then calculated (Fig. 4A). Compared with naïve mice (NT group), mice exposed to DSS and orally treated with PBS (PBS group) exhibited significantly higher DAI scores (Fig. 4B), resulting from decreases in body weight and increases in the scores of stool consistency and fecal bleeding (data not shown). Similar results were observed in colitis mice that were given oral NZ-VC (VC group). On the other hand, all macroscopic symptoms were suppressed by oral treatment with NZ-HO (HO group), and the DAI score on day 8 was significantly lower than the score of the VC group.

We also measured the colon length of each mouse on day 8 as a marker of inflammatory tissue injury (Fig. 4C, D). The colon from the PBS and VC groups was significantly shorter than that of the NT group. However, the HO group showed a significant improvement in colon length compared with the VC group.

Next, we performed a histopathological analysis of the colon on day 8. Colon sections were stained with hematoxylin and eosin (HE), and a histological score was determined following observation with light microscopy. No inflammatory signs or infiltration of inflammatory cells were observed in mice of the NT group, and a normal colon structure was seen (Fig. 4F). These results gave a histological score of 0 points for the NT group (Fig. 4E). On the other hand, almost all mice in the PBS group showed infiltration of inflammatory cells into the submucosa (6/7) and focal formation of mucosal erosion and ulceration (5/7) (Fig. 4G). In addition, several mice appeared to have severe tissue damage characterized by extensive pathogenesis (2/7). Oral treatment with NZ-VC did not improve these histological symptoms (Fig. 4H). Therefore, the histological scores of the PBS and VC groups were remarkably increased compared with the NT group (Fig. 4E). However, the histological score of the HO group was significantly lower compared to the VC group (Fig. 4E). HO mice showed inflammatory cell infiltration into the submucosa (3/7), and focal formation of inflammatory lesions (2/7) was present in only half or fewer mice in the PBS and VC groups (Fig. 4I). Moreover, no mice with severe tissue damage were observed in the HO group, and several mice showed no tissue damage (3/7).

### NZ-HO increases interleukin (IL)-10 and reduces pro-inflammatory cytokines

**Fig. 5:**
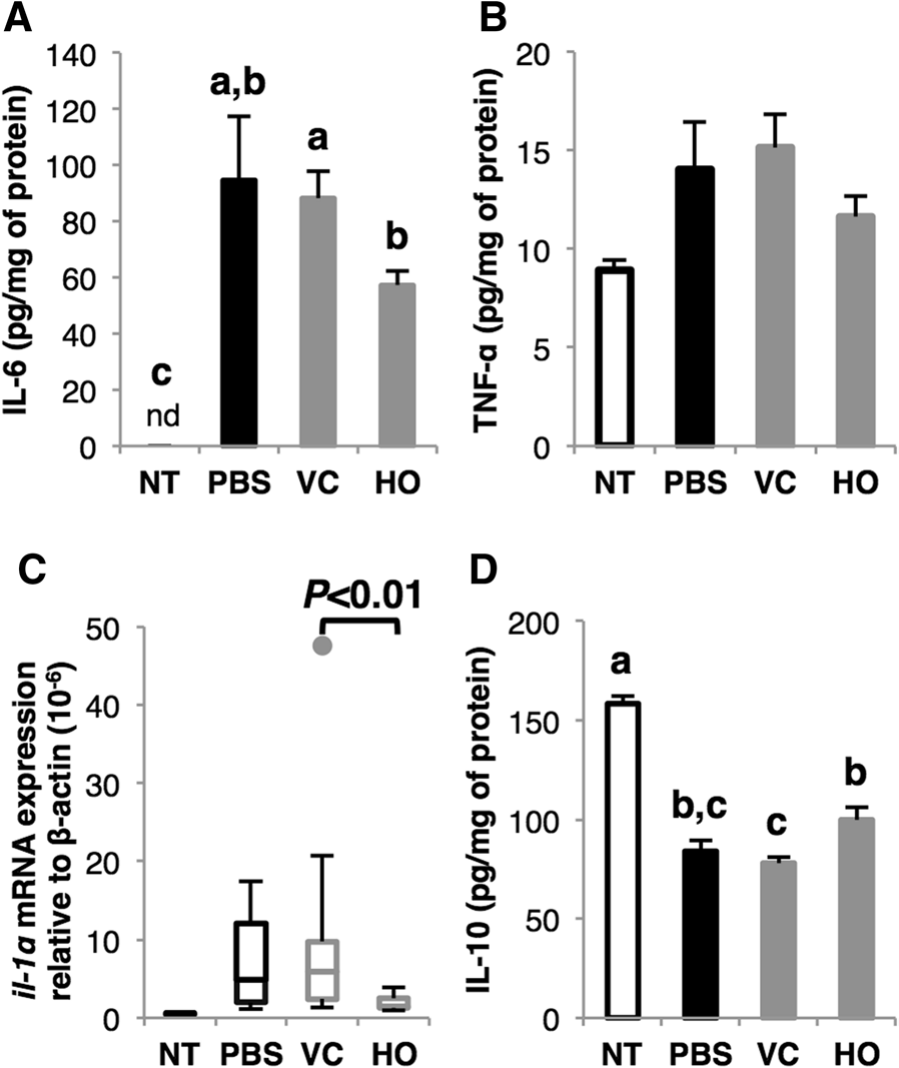
The modulating effect of NZ-HO on the expression of cytokines in colonic tissue. On day 8, protein extracts and total RNA were prepared from the middle and proximal colons, respectively. Protein levels of IL-6 (**A**), TNF-α (**B**), and IL-10 (**D**), and the mRNA expression of IL-1α (**C**) were analyzed with ELISA (**A**, **B**, **D**) or real-time qPCR (**C**). Data from ELISA are the mean + SE (*n* = 5–10). Values for the real-time qPCR analysis are shown as a *box plot* (*n* = 5–10). *Letters* (*i.e., a, b, c*) represent significant differences (*P* < 0.05). *nd* not detected

To investigate the molecular mechanisms of the anti-inflammatory effects of NZ-HO, we analyzed cytokine expression in the colonic tissue from mice on day 8. Protein levels of IL-6 (Fig. 5A) and tumor necrosis factor-α (TNF-α) (Fig. 5B), and mRNA expression of IL-1α (Fig. 5C) were measured as pro-inflammatory cytokines. The PBS group showed markedly higher IL-6 protein and IL-1α mRNA levels than the NT group (Fig. 5A, C). Similar to the PBS group, the VC group also showed high expression levels of IL-6 and IL-1α. The expression levels of IL-6 protein and IL-1α mRNA were significantly lower in mice of the HO group compared to the VC group. Regarding TNF-α protein levels, no significant difference was detected between the NT and PBS groups (Fig. 5B). Next, we validated IL-10 protein levels as an anti-inflammatory cytokine (Fig. 5D). In contrast to pro-inflammatory cytokines, the IL-10 level in the PBS and VC groups was similarly reduced compared to that of the NT group. The expression level of IL-10 protein in the HO group was significantly higher than that in the VC group.

## Discussion

We developed NZ-HO as a delivery vehicle that secretes bioactive rmHO-1. HO catalyzes the stepwise degradation of heme using molecular O_2_ and electrons provided by NADPH-cytochrome P450 reductase, and leads to generation of biliverdin with free iron and CO as final products [14]. This reaction process has been characterized in various spectroscopic studies with the use of the in vitro reconstructed HO reaction system [25, 26, 28–35]. In this study, we examined the bioactivity of rmHO-1 in this system using ascorbic acid as a reducing agent in place of NADPH-cytochrome P450 reductase. The HO reaction initially forms a heme-HO complex, and this process occurs readily in the reaction mixture independent of a reducing agent [25]. A previous study showed that the absorption peak of the heme-HO complex (405 nm) is slightly higher than the absorption peak of free heme (402 nm) [26]. In accordance with previous observations, we found a similar change in the solutions from the starting point of the reaction in which the absorption peak in the Soret region from a mixture containing the NZ-HO extract was slightly higher than the absorption peak from buffer or mixtures containing NZ-VC. The absorbance at 405 nm was greatly decreased in the solution containing NZ-HO extract at 30 min after the start of the reaction. The final product from the mixture containing NZ-HO exhibited a small peak around 400 nm. Yoshida and Kikuchi reported that the heme-HO complex is degraded to an Fe^3+^-biliverdin complex, which is a precursor of biliverdin [29], rather than iron-free biliverdin when ascorbic acid is used in this system [25]. Importantly, the Fe^3+^-biliverdin complex exhibits an absorption spectrum that is similar to the result obtained in our study. Our results show that rmHO-1 formed an enzyme-substrate complex and produced the Fe^3+^-biliverdin-HO complex in the presence of ascorbic acid. We concluded that rmHO-1 produced by NZ-HO is active.

Several published reports have described methods for oral delivery of gmLAB to the intestinal tract [36, 37]. Steidler et al. successfully developed a gmLAB that secretes the bioactive anti-inflammatory cytokine IL-10 (LL-mIL-10), and demonstrated that daily oral administration of LL-mIL-10 reduces DSS-induced colitis in mice [10]. More recently, Motta et al. showed that an *L. lactis* strain that secretes Elafin, an endogenous serine protease inhibitor with a wide range of anti-inflammatory properties, protects various IBD model mice and cultured human intestinal cells from inflammatory insults and restores homeostasis [9]. In this study, we examined the accessibility of NZ-HO to the colon and rmHO-1 expression in the colonic mucosa in healthy and DSS-treated colitis mice. Viable NZ-HO clearly reached the colon following oral administration in an assay using GM17 cm plates and colony-direct PCR. To examine the biodistribution of rmHO-1, we performed immunohistochemical analysis with an anti-His-tag Ab to distinguish exogenous mHO-1 from the endogenous protein. NZ-HO secreted rmHO-1 onto the mucosal surface of the colon, and secreted rmHO-1 diffused into the mucosal epithelial cells, the crypt, and the lamina propria. In NZ-VC administered group, we also observed emergence of colonies on the GM17 cm plate and positive immunohistochemical reactions. NZ-VC expresses chloramphenicol acetyltransferase resistance with pNZ8148#2:SEC. Colony-direct PCR showed that colonies obtained from the NZ-VC administered group included an empty vector. Sequencing analysis showed that the amplified DNAs were identical to the vector sequences. These results suggest that the positive immunohistochemical reactions with the anti-His-tag Ab observed in the NZ-VC administered group indicate expression of a His-tagged peptide from an empty vector.

Recently, the number of IBD patients has been increasing in Western countries as well as Japan, and novel therapeutic strategies are needed. Here, we demonstrated that NZ-HO improved DSS-induced acute colitis in mice. Daily oral administration of NZ-HO significantly reduced IBD-like symptoms as demonstrated by a decrease in the DAI score, partially reversed colon shortening, and improved inflammation-related histological changes compared with NZ-VC-treated mice. HO-1 exerts a wide range of anti-inflammatory and cytoprotective activities through generation of heme catabolites [13]. Our results showed that IL-10 production in the colon of NZ-HO-treated mice was significantly higher than in mice given NZ-VC. The strong relationship between IL-10 and HO-1 has been established in previous studies. IL-10 is one of the most important anti-inflammatory cytokines, and its signaling pathway is essential for maintaining mucosal immune homeostasis [38]. In fact, IL-10- and IL-10 receptor-deficient mice develop spontaneous enterocolitis [39, 40]. In monocytes/macrophages, IL-10 and HO-1 form a positive feedback circuit, which depends on the activation of a p38 mitogen-activated protein kinase pathway by CO derived from the HO-1 reaction and phosphorylation of signal transducer and activator of transcription 3 to amplify the anti-inflammatory signal [41–43].

Zhang et al. reported that HO-1 increases regulatory T cells, which are a major IL-10-producing cell population, rather than IBD pathogenic T helper 17 (Th17) cells via blocking IL-6/IL-6 receptor signaling in mice with DSS-induced colitis [24]. Pro-inflammatory cytokines including IL-1, IL-6, and TNF-α are produced following massive infiltration of immune cells in inflammatory lesions, and play a critical role in the pathogenesis of IBD. In particular, IL-6 directly affects the differentiation of Th17 cells, which constitute a specialized helper T cell subset, and exacerbates inflammation via production of Th17 cytokines. IL-6 is also critically involved in T cell-mediated inflammatory responses in IBD [44]. In this study, we observed dramatic inflammatory cell infiltration into the colonic lamina propria and a remarkable increase in IL-1α mRNA expression and IL-6 protein production in our model. Importantly, these increases in pro-inflammatory cytokines were significantly suppressed by oral administration of NZ-HO. Because the catabolites generated by the HO reaction exert various bioactivities [13], NZ-HO not only affects the immune system but may also impact other physiological systems.

Biliverdin and its subsequent metabolite bilirubin exert anti-oxidative properties by scavenging peroxyl radicals and inhibiting lipid peroxidation, and can mediate anti-inflammatory and anti-apoptotic effects of HO-1 [45]. Berberant et al. demonstrated that intraperitoneal injection of exogenous bilirubin in mice ameliorates DSS-induced acute colitis [46]. CO has an anti-apoptotic effect [47] as well as the above-mentioned immunomodulatory property. The beneficial effects of NZ-HO may be due to multiple mechanisms of action including physiological and immunological effects such as up-regulation of IL-10. Further investigations of the detailed mechanisms are needed to confirm that NZ-HO is useful as a mucosal therapeutic agent.

## Conclusions

In conclusion, we demonstrated three major points in this paper: (1) we successfully constructed a gm-*L. lactis* strain that secretes bioactive rmHO-1; (2) viable NZ-HO was directly delivered to the colon via oral administration, and rmHO-1 was secreted onto the colonic mucosa in mice; (3) daily oral administration of NZ-HO in mice alleviated DSS-induced acute colitis accompanied by macroscopic and histopathological changes, and beneficially modulated the expression of cytokines in colonic tissue. These results suggest that NZ-HO as a mucosal therapeutic agent may be an attractive candidate for treating IBD.

## Methods

### Bacterial strains and growth conditions

*L. lactis* NZ9000 (NZ9000; MoBiTec, Goettingen, Germany) was used as a host strain and was grown anaerobically at 30 °C in M17 broth (BD Difico™, Becton, Dickinson and Company, Sparks, MD, USA) supplemented with 0.5 % glucose (GM17). Genetically modified strains of NZ9000 (gmNZ9000) were grown in GM17 with 10 μg/mL chloramphenicol (GM17 cm).

### Construction of gmNZ9000

A gene coding for mouse HO-1 (mHO-1, GenBank accession number: NM 010442.2) was synthesized by Eurofins Genomics (Tokyo, Japan) with optimized codon usage for *L. lactis*, and was then cloned into the lactococcal secretion plasmid, pNZ8148#2:SEC (Fig. 1a) [48]. The resulting mHO-1 secretion vector was electroporated into NZ9000 with a Gene Pulser Xcell electroporation system (Bio-Rad Laboratories, Inc., Hercules, CA, USA), generating NZ-HO. NZ9000 was also electroporated with an empty plasmid to generate a vector control strain (NZ-VC).

### Nisin induction and detection of rmHO-1

All experimental procedures in this section were performed according to previously described methods [48, 49]. Briefly, gene expression in gmNZ9000 was induced with 1.25 ng/mL nisin (MoBiTec) for 3–4 h in 2-mL cultures. Bacterial cells and supernatants were separated by centrifugation, and protein samples were prepared. These samples were subjected to sodium dodecyl sulfate–polyacrylamide gel electrophoresis (10 % (*v*/*v*) polyacrylamide). Electrophoresed proteins were visualized by gel staining with Coomassie Brilliant Blue or transferred from the gel onto a polyvinylidene difluoride membrane for western blotting. Western blotting was performed with mouse anti-His-tag Ab (1/1000) (652501; BioLegend, San Diego, CA, USA) or rabbit anti-HO-1 Ab (1/1000) (SAB2101053; Sigma-Aldrich, St. Louis, MO, USA), followed by incubation with horseradish peroxidase (HRP)-conjugated goat anti-mouse IgG Ab (1/5000) (A4416; Sigma-Aldrich) or HRP-conjugated goat anti-rabbit IgG Ab (1/5000) (A0545; Sigma-Aldrich). The experiments were repeated three times.

### Quantification of mHO-1 in gmNZ9000

Nisin-induced gmNZ9000 cells were prepared as described above. Cells were washed once with ice-cold PBS and resuspended at OD_600_ = 1 in 200 μL PBS with protease inhibitor cocktail (Roche Diagnostics, Indianapolis, IN, USA). Bacterial cells were then disrupted using a beads beater (Beads Crasher μT-12, Taitec Corporation, Saitama, Japan) with glass beads. Soluble fractions were collected by centrifugation and stored at −80 °C. The mHO-1 concentrations in the cellular samples from six independent experiments were quantified using an ELISA Kit (Heme Oxygenase 1 Mouse SimpleStep ELISA Kit, Abcam, Cambridge, UK) according to the manufacturer’s instructions.

### Bioactivity assay of rmHO-1

Expression of the HO-1 gene in gmNZ9000 was induced as described above. Cells were harvested from 50-mL cultures by centrifugation and washed twice with ice-cold 50 mM potassium phosphate (KPi) buffer (pH 7.4). Cells were resuspended at OD_600_ = 1 in 650 μL Kpi buffer. Cellular extracts were prepared using a beads beater as described above and immediately used in the following experiment. An HO-1 activity assay was performed as described [25, 29]. Briefly, the reaction mixtures contained 0.5 mM sodium ascorbate, 0.25 % Tween20, 500 μL cellular extracts from gmNZ9000, and 50 mM Kpi buffer (total volume: 1960 μL). The reaction was initiated by the addition of 0.2 μM heme (Sigma-Aldrich) (final volume: 2 mL), and the mixtures were incubated at 23 °C for 30 min. Kpi buffer (500 μL) was used as the buffer control in place of cellular extracts. The UV–VIS-NIR spectra from 250 to 1000 nm were monitored with a UV–VIS spectrophotometer (model UV-1800; Shimadzu, Kyoto, Japan) at 0 and 30 min. The experiments were repeated three times.

### Mice and ethics

Female C57BL/6 mice (7 weeks of age) were purchased from Japan SLC (Shizuoka, Japan), housed under temperature- and light-controlled conditions, and fed a standard diet (MF, Oriental Yeast Co., LTD., Tokyo, Japan) and sterile water or sterile water containing 3 % DSS ad libitum. Mice were used for experiments after preliminary housing for 2 weeks (9 weeks of age, 20 ± 2 g). All experimental procedures were carried out in accordance with the Regulations for Animal Experimentation of Shinshu University, and the animal protocol was approved by the Committee for Animal Experiments of Shinshu University. Based on national regulations and guidelines according to Law No. 105 and Notification No. 6, all experimental procedures were reviewed by the Committee for Animal Experiments and finally approved as No. 240078 by the president of Shinshu University.

### Protocol for inducing colitis and method of oral administration

DSS-induced acute colitis in mice is widely used as a simple and well-characterized model of human IBD [50, 51]. A schematic of the schedule of the experimental procedure is shown in Fig. 4A. After preliminary housing, mice were divided into four groups: NT group (*n* = 5, naïve), PBS group (*n* = 10, DSS treated, PBS treated), VC group (*n* = 12, DSS treated, NZ-VC treated), and HO group (*n* = 12, DSS treated, NZ-HO treated). To induce colitis, mice freely drank sterile water containing 3 % DSS (MW = 36,000-50,000 Da; MP Biomedicals, LLC, Solon, OH, USA) for 6 consecutive days (days 0–5). On day 5, the bottle contents were changed from DSS water to sterile water, and mice were allowed to drink water ad libitum until the last day of the experiment (day 8). Mice in the NT group only drank sterile water. We monitored drinking volumes throughout the experimental period and confirmed that all mouse groups consumed similar volumes (4 ± 2 mL/day) of water regardless of the presence or absence of DSS (data not shown). For oral administration, gene expression was induced in a 50-mL culture of gmNZ9000 as described above. Cells were harvested, washed twice with PBS, and resuspended in PBS at 2.5 × 10^10^ CFU/mL. The bacterial suspension (200 μL, corresponding to 5 × 10^9^ CFU) was immediately administered intragastrically (i.g.) to mice in the VC and HO groups, once daily beginning 2 days before the start of DSS exposure (day −2) until the last day of DSS administration (day 5). Mice in the PBS group were administered 200 μL PBS in place of the bacterial suspension. On day 8, mice were euthanized by cervical dislocation. The colon (i.e., just under the cecum to the anus) was immediately collected, the luminal contents removed, and the length was measured. The colon was divided equally into three segments and stored at −80 °C.

### Evaluation of inflammatory severity

To determine the macroscopic severity of inflammation, body weight loss, fecal bleeding, and stool consistency were monitored daily after DSS exposure in all mice. These three parameters were graded on a scale of 0–4 as follows: body weight loss (0, ≤5 %; 1, 5–10 %; 2, 10–15 %; 3, 15–20 %; 4, >20 %), fecal bleeding (0, negative; 2, slight; 3, moderate; 4, gross), stool consistency (0, normal; 2, loose; 4, diarrhea). The sum of the three scores was then calculated as the DAI score (maximum score = 12).

### Histopathology

The distal colons were taken from 5 to 7 mice showing the average DAI score for each group on day 8. Colonic tissues were fixed with phosphate-buffered 4 % paraformaldehyde, embedded in paraffin wax, sliced, and stained with HE [52, 53]. Histological pathology was evaluated under light microscopy using a scoring system to evaluate inflammatory cell infiltration and tissue damage [54].

### Cytokine ELISA

Crude protein extracts from the middle section of colons were prepared by slight modification of previously described methods [55]. Briefly, tissues were homogenized using a beads beater, and the supernatants were collected. Protein concentrations were measured with a BCA Protein Assay Kit (Thermo Scientific, Rockford, IL, USA) and adjusted to 2.5 or 5 mg/mL by dilution with lysis buffer to determine the concentrations of IL-10 and TNF-α or IL-6, respectively. Cytokine concentrations in the crude protein extract from 5 to 10 mice with the average DAI score for each group were quantified using an ELISA Kit (IL-6 and IL-10, eBioscience Inc., San Diego, CA, USA; TNF-α, Diaclone SAS, Besancon Cedex, France) according to the manufacturer’s instructions.

### Real-time quantitative PCR

Total RNA from the proximal colons of 5-10 mice with the average DAI score for each group on day 8 was isolated with a High Pure RNA Tissue Kit (Roche Diagnostics) according to the manufacturer’s instructions. Then, cDNA was synthesized using a PrimeScript RT reagent Kit (Takara Bio Inc., Shiga, Japan) according to the manufacturer’s instructions. Real-time qPCR analysis was performed with SYBR Premix Ex Taq (TaKaRa Bio) using primers specific for mβ-actin and mIL-1α (TaKaRa Bio) [52, 56].

### Survival of NZ-HO in the mouse intestine

Acute colitis was induced in mice by 6 days of drinking 3 % DSS in the drinking water. In the healthy group, mice drank distilled water during the induction phase of acute colitis. The analysis of intestinal accessibility of gmLAB was performed as previously described [57, 58]. Briefly, on the day following the treatment, mice were given 10 serial i.g. doses of 200 μL PBS or the NZ-HO suspension (corresponding to 5 × 10^9^ CFU) once every 30 min. One hour after the final administration, the colon was extirpated from euthanized mice. To analyze the retention of NZ-HO in the colon, the entire colon (including luminal contents) was homogenized in PBS containing 1 % fetal calf serum. The homogenates (100 μL of 10^5^-fold dilutions) were plated on GM17 cm agar plates, which were incubated anaerobically at 30 °C. After 2 days, plates were observed, and photos were taken with a digital camera. To detect rmHO-1, colonic tissues were lightly fixed with 4 % paraformaldehyde, embedded in Tissue-Tek O.C.T. compound (Sakura Finetek, Tokyo, Japan), frozen in liquid nitrogen, and sectioned. Immunohistochemistry was then performed with rabbit anti-6 × His-tag Ab (1/100) (ab9108; Abcam) [59] followed by incubation with biotinylated goat anti-rabbit IgG Ab (1/300) (AP132B; Millipore, Billerica, MA, USA) [60]. These sections were further treated with HRP-conjugated streptavidin (1/300) (474-3000; KPL, Gaithersburg, MD, USA), and the signal was visualized with 3,3′-diaminobenzidine.

### Colony-direct PCR

Colony-direct PCR analysis of gmLAB was performed as previously described [61]. PCR amplification was performed in a 20-µL volume containing 10 µL of 2 × EmeraldAmp PCR Master Mix (TaKaRa Bio) and 0.5 µM each primer. The primers were as follows: *L. lactis* subsp. *cremoris*-specific primer pair [27] (forward (CreF): 5′-GTGCTTGCACCGATTTGAA-3′; reverse (LacreR): 5′-GGGATCATCTTTGAGTGAT-3′), pNZ8148-specific primer pair (forward (pNZ F3126): 5′-TGCCCCGTTAGTTGAAGAAG-3′; reverse (pNZ R340): 5′-TCAATCAAAGCAACACGTGC-3′). The PCR cycling conditions were: 95 °C for 5 min, followed by 40 cycles of denaturation at 95 °C for 30  s, annealing at 45 °C (CreF-LacreR) or 60 °C (pNZ F3126-pNZ R340) for 30 sec, and extension at 72 °C for 1 min. The PCR products (10 µL) were examined by 1.0 % (*w*/*v*) agarose gel electrophoresis. DNA was stained with ethidium bromide and photographed with a gel imager (Gel Doc EZ Imager, Bio-Rad Laboratories). DNA sequences of PCR products were determined by DNA sequencing analysis, which was performed by Eurofins Genomics. Homology analysis was performed by GENETYX-MAC (Genetyx, Tokyo, Japan) for the sequences of *L. lactis* NZ9000 (GenBank accession number: CP002094.1), pNZ8148#2:SEC, or pNZ8148#2:SEC-mHO-1.

### Statistical analysis

All statistical analyses were performed using a statistical software package (ystat2004.xls, Igakutosho Shuppan, Tokyo, Japan). One-way ANOVA followed by the Tukey–Kramer method was used to determine the significance of the differences in all experiments except for the real-time qPCR analysis, which was analyzed with the Mann–Whitney *U*-test. Differences were considered significant at *P* < 0.05. Values for the real-time qPCR analysis are expressed as a box plot. Values for quantification of the mHO-1 concentration are expressed as the mean ± standard deviation (SD). Other values are expressed as the mean + standard error (SE).
